# Professional perspectives on facilitators and barriers for high quality provision of health, education and social care services to disabled children in England during the COVID-19 pandemic: a qualitative study

**DOI:** 10.1136/bmjopen-2024-085143

**Published:** 2024-08-24

**Authors:** Hannah Merrick, Helen Driver, Chloe Main, Lily Potts, Siân Russell, Catherine Exley, Amanda Allard, Christopher Morris, Jeremy R Parr, Lindsay Pennington, On behalf of Resetting Services Team, Dawn Teare

**Affiliations:** 1 Population Health Sciences Institute, Newcastle University, Newcastle upon Tyne, UK; 2 School of Psychology, Newcastle University, Newcastle upon Tyne, UK; 3 Council for Disabled Children, London, UK; 4 National Children’s Bureau, London, UK; 5 PenCRU, University of Exeter Medical School, University of Exeter, Exeter, UK; 6 Cumbria Northumberland Tyne and Wear NHS Foundation Trust, Newcastle upon Tyne, UK; 7 Great North Children's Hospital, Newcastle upon Tyne Hospitals NHS Foundation Trust, Newcastle Upon Tyne, UK

**Keywords:** COVID-19, developmental neurology and neurodisability, health services

## Abstract

**Abstract:**

**Objectives:**

To understand how health, education and social care services for disabled children changed during the COVID-19 pandemic, what did or did not work well and what the impacts of service changes were on both professionals and families.

**Design:**

Qualitative study using semistructured interviews.

**Setting:**

Telephone and video call interviews and focus groups with professionals working in one of five local authority areas in England.

**Participants:**

78 health, education and social care professionals working with children in one of five local authority areas in England.

**Results:**

There was a significant disruption to services and reduced contact with families during the early stages of the pandemic; nevertheless, professionals were able to reflect on innovative ways they interacted with and sought to support and maintain health, education and social care provision to disabled children and their families. As waitlists have substantially increased, this and the longevity of the pandemic were perceived to have had negative consequences for staff health and well-being, the health and psychosocial outcomes of children and young people, and their parent carers.

**Conclusions:**

Key learning from this study for service recovery and planning for future emergencies is the need to be able to identify disabled children, classify their level of need and risk, assess the impact of loss of services and maintain clear communication across services to meet the needs of disabled children. Finally, services need to work collaboratively with families to develop child-centred care to strengthen resilience during service disruption.

**STRENGTHS AND LIMITATIONS OF THIS STUDY**

- Recruiting in five diverse local authority areas provided the opportunity to learn from a wide range of changes and experiences of changes.
- Qualitative methodology enabled in-depth data collection about reflections on service changes and decision-making.
- Including health, education and social care professionals provided a broad understanding of experiences and impacts of service changes.
- Framework analysis enabled inductive thematic analysis and data exploration while simultaneously maintaining a transparent audit trail.
- Potential response biases to participating in this study mean our findings may not reflect the experiences and perspectives of all professionals.

## Introduction

 The COVID-19 pandemic saw countries apply various measures and restrictions to limit virus spread. In the UK, on 23 March 2020, the Coronavirus Act passed into legislation and a national lockdown was implemented to slow the spread of the COVID-19 pandemic.^1^ Additional instructions were given to specific groups deemed to be clinically vulnerable to stay ‘shielded’ in their homes. Keyworkers attended work, but others did not. Schools were closed, except for the children of keyworkers and vulnerable children, for example, children with an Education, Health and Care plan (EHCP), with a child protection plan, receiving social care services or who would have difficulty accessing remote education.^2^ These measures interrupted access to healthcare, in-person learning, social and community services, networks and recreational activities. For disabled children and their families, this made access to activities, resources and support challenging. The support for disabled children often involves several agencies, and the quality of the support families receive can depend on good multiagency assessment, care planning and intervention.^3^ Where they existed, strong local support structures played an important role in facilitating good local decision-making during COVID-19 for disabled children.^4^

In the UK National Health Service (NHS), many health professionals were redeployed, elective hospital admissions and procedures were postponed or cancelled to prioritise critical care services and outpatient appointments were cancelled or offered as online appointments to reduce COVID-19 spread.^5 6^ During the pandemic, health professionals including doctors, nurses and allied health professionals worked under extremely challenging conditions and negative impacts on their health and well-being have been noted.^7 8^

Children’s education and social care services changed rapidly to primarily remote delivery and were focused on managing resilience and individual risks.^9 10^ In May 2020, in England, children with EHCPs (that define legally the services children must receive) found their rights formally downgraded by the Coronavirus Act 2020.^11 12^ Essential services outlined in EHCPs including mental health support, speech and language therapy, short breaks and one-to-one educational support were widely discontinued.^13^ Local authorities had to balance the challenges of keeping their workforce safe, responding to the needs of families and children to try to ensure children were safe and adapting to new ways of working. In combination, these changes substantially disrupted the delivery of services to disabled children and resulted in many children experiencing a partial or full loss of services for a sustained period, placing considerable strain on families.^14^

The Resetting Services to Disabled Children programme of research aimed to learn from the pandemic to understand how services could be delivered better to provide high-quality care to disabled children and their families in times of emergency and as the UK NHS is remodelled. This paper reports on analyses of interviews carried out with health, education and social care professionals working with disabled children and young people to understand how services changed during the COVID-19 pandemic, what worked well and what the impact of service changes was on both professionals and families. The perspectives of parent carers on service changes during the pandemic are reported in a parallel paper.^15^

## Methods

### Participants and recruitment

Eligible participants were health, education or social care professionals (early years provider, occupational therapist, physiotherapist, speech and language therapist, paediatrician, neurodisability consultant, psychologist, teacher, social worker and a third sector worker where appropriate), managers and commissioners. Participants were recruited from five diverse local authority areas in England, chosen to be diverse according to geographical (eg, urban and rural) and demographic (eg, level of deprivation) characteristics and the number of organisations providing statutory services to disabled children. We aimed to purposively sample approximately 15 multidisciplinary professionals per area to allow us to capture a range of different views. Purposive sampling was used to ensure a range of perspectives were captured. A local ambassador in each of the five sites was provided with information about the study to cascade to their peers (eg, through in-person discussion and emails). Interested professionals registered via a link to an online survey on the study webpage or emailed the research team directly and were then contacted by email and provided with an information sheet. All participants documented consent prior to the interview with an electronic or typewritten signature on the consent form.

### Interview procedures

Semistructured interviews with individuals and/or focus groups were conducted between November 2021 and September 2022. Focus groups included professionals working in the same area or team. Interviews and focus groups were conducted by videoconferencing (via Teams/Zoom). At the start of the interview, participants provided additional verbal consent to their participation and recording of the interview. Given the sensitive nature of the interview and the topics being discussed, it was explained to all participants that they could stop the interview at any point if they wanted to and did not need to answer a question if they did not wish to. All interviews were transcribed verbatim during the interview using Microsoft Stream for Teams or Zoom. Transcripts were checked for accuracy, corrected, and anonymised.

The interview topic guide included questions focusing on (1) how and in what ways services changed; (2) the impact of changes on both families and professionals; (3) what worked well, what did not work well and for which groups different approaches worked better or worse; (4) which characteristics defined high-quality experience during the pandemic; (5) which factors supported optimal delivery of services during the pandemic and (6) what would have made substantive differences to improve the experience of families during the pandemic (see online supplemental material 1).

### Analysis

Data collection and analysis were iterative, following the principles of the constant comparison method.^16^ This systematic iterative approach allowed us to quickly capture the emergent themes and explore those with the research team and our patient and public involvement (PPI) advisors.^17^ Two researchers (HM and HD) and psychology students (Chl M and Lil P) analysed the anonymised transcripts. Analysis was informed by the framework approach.^18^ An analytical framework was developed through open coding of initial transcripts and with reference to the Effective Practice and Organisation of Care taxonomy (EPOC)^19^ for service change codes. Data were charted using the framework matrix and then summarised by category.^20^

### Reflexivity

Analysis and interpretation were led by coders HM and HD and supported by Lindsay P, CE and the wider research team. HM has no lived experience of neurodisability or having a family member with neurodisability. HD is a parent carer of a young person with neurodisability but not situated in any of the local authority areas included in this study. Researchers were conscientious in reporting the narrative of participants and engaging in reflection and discussion to ensure accurate interpretation of the interview findings. Coding and framework analyses have been shared with the wider research team drawing in clinical and qualitative perspectives. The varied experience and diverse positioning of the research team and PPI engagement established trustworthiness in the research process.

### Patient and public involvement

Public and patient involvement and engagement informed each stage of the study. Research design involved collaboration with parent carers, children and young people with neurodisability, and representatives of advocate organisations; Parent Carer Forum, PenCRU (Peninsula Childhood Research Unit) and the Council for Disabled Children (See online supplemental table 1).

## Results

78 professionals were interviewed between November 2021 and September 2022 through 49 individual interviews and nine focus groups (n=29, up to six participants per group). Participants included healthcare providers, social care professionals, ‘organisers and funders of services’ (commissioners) and education professionals (see table 1).

**Table 1:** Participant's professional roles

Role	N	Area 1	Area 2	Area 3	Area 4	Area 5
Commissioning/management						
Commissioner	6	1		1	2	2
Early years lead	5		3			2
Designated medical officer/designated clinical officer (DMO/DCO)	5	2	2	1		
Head of SEND/SEND lead	4	1	1		1	1
Therapies lead	4		2	1	1	
Social care lead	2		1		1	
Healthcare professionals						
Paediatrician	6	2			3	1
Epilepsy nurse specialist	2	1				1
Paediatric surgeon	2					2
Physiotherapist	5	1			2	2
Occupational therapist	5			1	4	
Speech and language therapist	4		1	1	2	
Dietician	3	1			2	
Community nurse	3				1	2
Clinical psychologist	2		1	1		
Equipment services	2			1	1	
Health visitor	1		1			
Orthoptist	1	1				
Therapy assistant	1					1
Education and social care						
Social worker/family planner	3		2			1
Disabled children’s team manager	4	1	2	1		
Headteacher	3	1	2			
SENCo	2	1			1	
Educational psychologist	2				1	1
Service manager	1	1				
**Total**	**78**	14	18	8	22	16

SENCoSpecial Educational Needs CoordinatorSENDSpecial Educational Needs and Disability

With respect to professionals’ experiences of service delivery changes and the impact of these changes, we identified three overarching themes: (1) service changes implemented, (2) impact of service delivery changes and (3) learning for future service provision and emergencies.

### Service changes implemented

#### Decisions on service changes

National legislation (ie, the Coronavirus Act 2020), regulations (eg, The Health Protection (Coronavirus, Restrictions (No 1)) and guidance (eg, COVID-19: guidance for maintaining services within health and care settings infection prevention and control recommendations) led to professionals making decisions about changes to local service delivery. These determined where and how professionals could continue to see children and young people throughout the stages of the pandemic. However, in the initial stage of the pandemic, regulations and guidance and consequent decisions about their implementation changed frequently. Moreover, changes to guidance were not always perceived to be clear and were often felt to be open to interpretation (box 1a). Furthermore, all participants described guidance from governing bodies on health, education or social care sectors as being inconsistent. This reduced health professionals’ access to children in school and limited activities that could be undertaken in school if they were not considered part of a school ‘bubble’ and used different personal protective equipment (PPE) to school staff, reducing face-to-face access to children (box 1b). There were also contradictions around which children should be in school. Commissioners and designated clinical officers/designated medical officers (DCO/DMO) consistently described challenges around supporting providers to understand national guidelines, mediate decision-making and find ways for professionals to continue to support the children and young people.

Box 1 **Quotations for theme: service changes implemented**

**Decisions on service changes**

- “I think one of the challenges was the sheer volume of information that was coming from central government, which was changing on a very regular basis and then trying to tease out what was relevant to services for disabled children for example and not wanting to miss things.” (Commissioner, A4_02)
- “Well and there were guidelines coming in from governing bodies like the CSP [Chartered Society of Physiotherapy] saying you're not allowed to do like this way. Music therapy was saying it a different way. Speech therapy was saying it different way. We as an organization had to have a fair and equitable approach to all our different therapists. Because music therapists, are, you know, are no more special people than speech therapists are. So, it was all that conflicting advice from, when we were doing our risk assessment, we were pulling it from so many different areas.” (Headteacher, A1_02)
- “I think within the provider organisations, they had the discretion to decide which of their services they felt were, and I think they were given criteria to work to that were about the essential, critical, noncritical and that kind of thing. And then if there were any sort of contentious decisions or anything that they felt they needed a sort of system wide assessment of, then we had the command structure established… So, if there were decisions that needed to be made about opening and closing services it would go through that command structure and be signed off by the directors.” (Commissioner, A3_01)
- “The agencies came together immediately and were starting to look at what information we knew about families to start assessing how much support would be needed, what were the risks, who are we going to prioritise. And from, again in terms of what worked well, as somebody who’s worked in this field for many, many, many years, we're always looking at how do we get to this final position of a single view for a child so that we’re communicating well together. And we're always faced with barriers around how we do that. And yet, at the point of a crisis, we can do it, and we effectively and appropriately share information together, that means that we agree who’s going to do what and how they're going to respond…” (Commissioner, A1_10)
- “And then we had some of our, we share estates with the GP surgery, and they were very quick to shut the doors on us and say, no, you're not, you're not coming in here at all.” (Early years pathway lead, A5_06: focus group)
- “Services were already at breaking point in terms of capacity, so if you've got a team of 15 people, even if only one or two of those people are isolating with COVID and off sick, your capacity to do the work is so much more limited. And a lot of health professionals were being seconded to deal with the COVID response, so that had a huge impact on the community delivery of services for disabled children as well.” (Social worker, A2_09)

**Communication of service delivery arrangements**

- “If I'm really honest with you, what was good was the leadership. So, the structures that were above me made me feel safe to be innovative in some of our solutions that we came up with, because again, it came down to us risk assessing, and while there weren't lovely streamlined prepared pathways we could follow, we could kind of find our way by risk assessing and having team meetings and as a group making decisions.” (AHP Lead, A2_04)
- “We had weekly meetings with [Specialist nursing team]. So they were the people that were providing obviously some of the packages and some of the respite. We also had, I provided them with an open door, the therapy teams, with an open door around those children where there was evolving risk.” (Commissioner, A3_07)
- “So things like strategy meetings, things like child in need meetings, Child Protection Conference is, you know, all that sort of stuff I have to say, I was very dubious as to what that was gonna look like, particularly with a couple of my very tricky child protection plan cases. Thinking how is this all going to work? And I just was amazed. I mean it works. And it’s fine, and it’s OK.” (Paediatrician, A1_07)
- “So after the Easter holiday, we decided that we'd, what I was doing was I was calling the very vulnerable ones and the ones that were on the community nurse case load. But the rest of them, the teachers were phoning and if there was anything medical, I said please, when you phone and doing your weekly catch up, say, is there anything that we can help you with? Is there any other, any medical worries, any health worries, anything that the special school Nurse can help you with and then handing that to me and I was then picking that up.” (Paediatrician, A5_03)
- “Yeah, I mean it [talking to parent carer forums], it’s helpful for me. It just gave us a bit of a sense check, temperature check, in terms of where parent carers we're at and I know it’s a very generalised kind of perspective isn't it, but it was helpful for me to kind of understand whether what we were doing was the right thing to be doing at that time?” (Service lead, A4_01)

**Service delivery arrangements**

- “And it was quite a sudden change where the lockdown happened and we were told that basically paediatric speech, language therapy services were considered a kind of non-essential at that time. And so, the service was pretty much shut down. So we had to call all of our clients, cancel all of our clients and say we will be back in touch once we know it’s happening.” (Speech and language therapist, A4_15)
- “So within our organisation, like for example OT and speech and language therapy all went non face to face. But we took the decision that that wasn't appropriate for us. And straight away we had children with complex disability who were having respiratory problems and being admitted to the ward. So we had a sort of dual response, where children were, where we did a risk assessment that they needed face to face physio, we sort of had two or three staff that we're risk assessed and who were happy to do face to face.” (Physiotherapist A1_14)
- “… but I think PPE has been a real hindrance. Particularly in the first lockdown, we had to wear, obviously a mask, a visor, gloves and an apron. It was just really difficult because with some of the cases the behaviours of concern can be quite high, and it just meant that I felt really uncomfortable being in the home, not being able to like always see properly if the visor was foggy or. I just felt really uncomfortable and being able to protect myself and the parents if it came to it and I think it was also really difficult for the children because they didn't really understand what it was, so some of them would think I'm the dentist, and if they're scared of the dentist and they'll be like, no, I don't want you in the house.” (Social worker, A2_06)
- “We looked to deliver services slightly differently from both a holiday club but also [support groups] perspective. What we developed was half term clubs, that we used in our children centres, which weren't open at the time. And we invited small cohort of young people to come along and do activities. So, it was the same principle. So you know providing parents with the break from the caring role and activity for the child and young person. But we were able to use the venues that we identified and do our own COVID risk assessments to make sure that they were COVID-safe.” (Social care lead, A4_01)
- “So it was a bit scary because we, we tried out a lot of things we've never tried before at a speed that. You know, like we didn't have the structures behind us to, to give us the authorities we needed, but the leadership above us was, was very strong and solid and so we felt supported to, to be innovative and to, to come up with solutions in a timely way.” (AHP lead, A2_04)
- “So for me as a social worker, being able to identify some of the sensitivities around what’s going on in those relationships that helps us navigate a good partnership working. I think that that’s been very challenging, because it’s lost through that 2D screen. And of course, from a family’s point of view and social workers point of view, I think that when you're visiting a family, you're using all your senses to understand what the experience is and we can see during the course of the two years, the impact of not having that as your first point of contact.” (Social worker, A1_10)
- “Yeah, I kind of felt like it was, it was like being asked to design and implement a whole service overnight really wasn't it? Because it was just so radically different from what we've been doing and the infrastructure for what we needed to do, just obviously wasn't there either. So all the things that everyone has already said in terms of kind of IT, ways of working and some of the information governance and you know, privacy and confidentiality that goes with that.” (Early years lead, A2_18)
- “We were doing assessment [using telehealth], so trying to do movement assessment, which was tricky because parents on their own with more than one child were doing video calls. And we were looking at children doing the stairs and moving around. And yes, there were moments where that didn't feel very safe with the parent trying to video their child moving away. So, then we had to kind of think about, OK, how do you set up the appointment so that the phone is static, and we can still see what’s going on? And I think as physios, we were saying you, well for the majority you can't do physio remotely, you've got to have hands on to tell whether someone’s got a neurological abnormality in muscle tone.” (Physiotherapist, A1_014)
- “And also, we've seen a huge rise in dissociative seizures which are non-epileptic seizures caused by mental health causing a physical seizure and we've seen issues and big issues with waiting lists for things like CAMHS which has hugely impacted on our patients. But also on us, because we're then left trying to plug that gap without skills and resources to be able to do so. So, we are not providing the best service to those patients because it’s not our remit and because we don’t have the skills or capacity to be able to do it. But when no one else is doing it, we have to do something.” (Nurse, A5_08)

Effective organisational and management structures were consistently described across teams and areas as vital for implementing national and local guidance within each sector efficiently. All the commissioners interviewed from each sector described the Gold, Silver and Bronze command (a command hierarchy used for major operations by the emergency services of the UK) which met on a frequent basis to review national guidance and make decisions on provision. In the main, staff thought these structures allowed for quick escalation and response to issues around the implementation of service delivery decisions. In individual teams across health, education and social care, many professionals referred to the positive impact of having strong and responsive leadership (box 1c). Professionals who worked within smaller teams said they felt they were able to interpret the guidance themselves to meet the needs of their specific service and take more initiative in how to continue to provide services. Bidirectional feedback between management structures and those working directly with families was important to allow for specialist knowledge to inform decision-making.

The most vital decisions being made were about which children and young people should be prioritised for treatment, have access to school and receive in-person care. All professionals working in each of the sectors described the first task being to risk assess their service users using Red-Amber-Green (RAG) ratings based on children’s and families’ levels of need. The majority said there was no clear guidance on how to RAG rate the needs of children and young people, but examples given by participants that led to a ‘red’ rating included ‘under 5s’, equipment needs, children who needed postural support or pain management, children who were changing medications, children with respiratory needs and families where there may be a safeguarding concern or child protection order in place. The introduction of the ‘Control of Patient Information’ (COPI) notice in June 2020 allowed service leads to share and collate RAG data across the sectors and feed information up through to senior teams in the locality to inform decisions about what provision was needed, level of risk and where resources needed to be focused (box 1d). Several of the service leads and commissioners expressed disappointment that the COPI notice was removed in June 2022 meaning the return of barriers to communicating and coordinating individualised care across services.

While RAG rating indicated who needed the support, commissioners’ and managers’ decisions on the provision of services were also influenced by the resources (human, financial and physical) available in each locality. Regulations, in particular ‘stay at home’ orders and social distancing measures, meant settings could not run at their normal capacities. In one area, health professionals described how clinic spaces were repurposed for COVID-19-specific care, in most areas community spaces were closed and as described above, school spaces were limited (box 1e). Senior leaders (eg, Gold command) made decisions to redeploy paediatric staff to services where more capacity was deemed to be needed (eg, adult services, COVID-19 wards or vaccination centres later in the pandemic), leaving children’s services with reduced capacity to maintain contact with families, leaving commissioners and service leads with reduced options on how to, and prioritising to whom they should, deliver care. The biggest impact of redeployment to other NHS services/departments was described for Allied Health Professionals (AHPs). There was variation across our participating sites on the proportion of AHPs redeployed and length of redeployment. In one area, plans for redeployment were not implemented, in another area redeployment was voluntary and in the remaining areas, AHP participants described their teams being reduced significantly (eg, one physiotherapy team was described as going from 10 to two members of staff) because of redeployment which decimated the capacity of that service. One AHP team stated redeployment and its impact on how care packages could be delivered compounded existing challenges to delivering the necessary support to families due to staff shortages prior to the pandemic (box 1f).

Health, education and social care professionals described trying to deliver as much as possible within the changing regulations and capacities and keeping families as informed as possible about service access. They also acknowledged the complex choices parent carers were making to protect their child and vulnerable family members. The headteachers and school nurses all observed that many parent carers were unsure about whether being at home or in school was the safest option for their child. Likewise, some health professionals spoke about their concern that families did not come forward with problems and access treatment because they were afraid of infection, and AHPs and social care professionals believed that some families were reluctant or resistant to returning to in-person appointments.

#### Communication of service delivery arrangements

Professionals across all sectors said they valued having an open dialogue with their leadership on how decisions on service delivery were being implemented. A factor facilitating continuing care was managers giving clear remits for what could and could not be done during the different stages of the pandemic and being receptive to feedback and innovative ideas (box 1g). Frontline staff found this especially helpful, given inconsistencies in guidance that led to confusion and frustration. Many health professionals described guidance being more focused on adult and acute settings, requiring greater interpretation for paediatric and community settings. All participants from all sectors reported more emails and meetings during the pandemic to ensure they were updated on decisions about service delivery and to bridge the gaps in the national and local guidance received (box 1h).

There were mixed opinions across the sectors on how cross-sector communication improved (or not) during the pandemic. Some education and social care professionals found communication and access to health professionals easier and better during this time. Participants from all sectors referred to how telehealth approaches had supported multidisciplinary team meetings during the pandemic and getting ‘the right people in the virtual room’ to make decisions and continue care (box 1i). Education professionals described mixed opinions on how telehealth supported communication across sectors. While attendance at multisector meetings around a family improved, it was also sometimes harder to contact other professionals if they worked remotely or were not in their usual locations to contact, for example, school staff trying to contact health service professionals or allied health professionals being able to contact equipment services.

In the initial stages of the pandemic, where capacity allowed, the majority of frontline staff described calling families to explain the service changes and check in on how families were coping. They tried to maintain this personal contact as much as possible, recognising a greater need to support families during lockdowns (box 1j). They reported using multiple modes of communication with families including emails, texts and phone calls to make sure there were open lines of communication on how their service was continuing to support children and young people.

Within all five areas, leads in each sector described sending newsletters to families and providers and holding webinars to communicate decisions to all providers, education settings and importantly their local Parent Carer Forums. One commissioner described a ‘hub’ run by redeployed education staff and supported by health and social care staff as a central point of contact and advice for schools and families. All the commissioners said they tried to be proactive in maintaining communication with families, including using apps, sending out newsletters via schools and holding regular Question and Answer sessions to keep families updated on service changes. This also allowed parent carers to feedback to commissioners on what they needed and how decisions were affecting them and their children (box 1k).

#### Service delivery arrangements

The most significant change in service delivery described was the stopping or substantial reduction of in-person appointments following the list of essential services by the government. Health and social care professionals said that the offer of in-person appointments was prioritised for specified activities determined by guidance from professional bodies and service management, but also based on their interpretation of guidance, availability of space and resources (box 1l). Those working in social care and early years education services described a push to continue, and return to, in-person appointments as soon as possible with the necessary risk assessments. Several of the paediatricians and AHPs described making team decisions to continue in-person appointments as soon as possible for new referrals, those with problems that required a physical assessment and those who had not engaged with telehealth (box 1m). Social care teams said they continued in-person child protection assessments using measures to do this as safely as possible (eg, masks and aprons). Processes for managing in-person hospital clinics were adapted, with families needing to wait in car parks, and less clinic space was available. Community healthcare teams and social care professionals said they continued home visits where necessary, meeting people in their gardens where possible by wearing the necessary PPE. However, the PPE needed for in-person appointments to be carried out safely was described as interfering with professionals’ typical interaction with families (box 1n).

A DCO and a DMO described coordinating with schools to move equipment from schools to family homes where possible so the child could continue therapy. Some AHP teams described hiring large community spaces that were not being used to be able to continue to provide services in a socially distanced space. Likewise, one social care service lead described using spaces and budgets differently to provide holiday clubs later in the pandemic (see box 1o). Participants described how the need for changes in service delivery as lockdowns and social distancing measures were extended led to some opportunities to develop and try new and innovative ways of seeing and supporting families when ‘usual care’ could not be delivered, for example, developing online resources, YouTube videos and using spaces differently (see box 1p).

In-person appointments/visits were rapidly replaced by increased use of telehealth. This was delivered first by telephone and later with video consultations (using systems such as Attend Anywhere and MS Teams). The most frequent barrier to telehealth described by nearly all participants was technical problems and lack of internet connection, particularly in more rural areas, which was potentially adding to existing inequalities in care. Social care, medical and allied health professionals felt the quality of the interaction with families was not as good when remote and it was more difficult to engage young people and hear their voice in the appointments. They described less ‘casual’ interaction with families and reduced ability to pick up on non-verbal cues and provide young people with a confidential space to discuss topics away from their parent carers. Social workers also highlighted the limits of telehealth compared with in-person visits as they were unable to fully understand the home environment (box 1q).

Despite such barriers, telehealth allowed the continuation of care and support during the pandemic. Some of the medical professionals described how telephone calls enabled more private and open conversations with parent carers without children being present. Health professionals agreed telehealth was more feasible with families already known to a service, with whom they had an existing relationship, compared with families newer to the service. The greatest challenges with telehealth were expressed by AHPs who needed to engage children in an appointment, for example, observing a child doing an activity, participating in an activity with a child or needing to physically examine a child. AHPs described learning a new skill set and approach for working online overnight (box 1r).

Medical and allied health professionals had mixed opinions on how feasible it was to carry out assessments using telehealth (box 1s). For many services, assessments stopped until they could be done in person, while others did what could be done online (eg, taking history, questionnaires and viewing movement online). A medical professional in one area also described how the global shift to telehealth meant they could attend training on carrying out autism and Attention Deficit Hyperactivity Disorder (ADHD) assessments via video consultation, which enabled them to give families the choice of taking part in an online assessment or waiting for an in-person assessment.

The limited access to families engendered by reduced workforce capacity (illness and redeployment) and social distancing regulations led to some professionals across all levels and sectors describing taking on wider roles and remits to ensure the monitoring and safety of children was maintained. For example, several nurses and AHPs described feeling like they were plugging gaps in other services when they were the only service in contact with a family (box 1t). Within education and social care, a few of the commissioners and education professionals described staff who could not carry out their usual roles, supporting families in other ways by providing outreach. For example, one social care lead described how support workers helped with food shopping and collected prescriptions for families, while a headteacher in another area described how teaching assistants visited homes to deliver school supplies.

### Impact of service delivery changes

Changes to service delivery were perceived to have had an adverse impact on care pathways, the workforce and the health and well-being of children and young people and their parent carers.

#### Care pathways

There were negative impacts described on the management of referrals and waitlists in allied health, medical and social care services (box 2a,b). In the initial stages of the pandemic, participants in each sector described a pause in the number of new referrals received while services were closed or running at a much-reduced capacity. However, referrals for social care, medical and allied health services were described as rapidly increasing with the reopening of schools and other services. Consequently, pre-existing long waitlists were exacerbated, for example, the autism assessment waiting list.

Box 2 **Quotations for theme: impact of service delivery changes**

**Care pathways**

- “It’s the backlog, the legacy that the two years that we've had to go through has left in terms of our services ability to respond quickly to children’s needs. We've got a massive backlog now of children waiting to be seen by our therapy services, for example. That’s going to be really difficult to overcome, and those children need our help.” (Speech and language therapist, A5_02)
- “ I think it makes you realise and that we need to, we need to catch up on this backlog. I don't really know how. We need to be more efficient because we can't catch up on the backlog and keep seeing new patients at a particular rate, we have to be a little bit sort of pragmatic about what do we need to see and catch up. Because long waits are awful for everybody and totally unacceptable. But happening. And you know the value of face to face new patient appointments cannot be underestimated. They have to, they have to stay.” (Dietician, A4_018)
- “And kind of less appropriate referrals. So it, also GP is not doing face to face appointments had a big impact on us. So we're quite often the first person that’s doing a face to face appointment and a lot of the time it’s kind of you know kind of whizzing through them to say yeah, everything’s OK, it’s giving some advice.” (Paediatrician, A1_14)
- “We've got another 8ish, who are 18 this year. We've got others who are 18 at the start of next year and then we've got about another 15 16-year-olds coming through, so we've got quite a big chunk of kids who are in that transition phase. And it is concerning. So I think there’s definitely been a knock on in terms of transition and it may well come up in your study as an area for parents because they start to think about transition when their child is 14–15. They start to think what happens when they're an adult and things like the marketplace events that transitions used to offer, which is where all the providers would come, the parent could physically talk to people and see what was on offer and hear about what my child might be able to access when they're 18, that all stopped.” (Social worker, A2_08)
- “The safeguarding we continued as per, but we upped it, so we did more visits, so we did, in between we had agreement to do some virtual visiting as well as full PPE visits in the family homes because obviously we had quite clinically vulnerable young people, it was around, how do we plan for those as well? So, because we had the laptops and we had the Internet, we did virtual visits. So it might be that a social worker was actually outside of the family home still. So, there would be one having a doorstep visit with the family, but there'd be someone walking around the family home with access to a mobile or, so we were doing virtual safeguarding visits as well by, show me your bedroom. Show me your cupboard. Show me everything you know. But we were there. We were outside the house while it was happening.” (Social worker, A3_06)

**Workforce well-being**

- “Uhm, I think there was also really valuable support in terms of staff well-being. You know, we're very well aware of the impact that the pandemic had on staff. And you know, there was that concern from a lot of people about just being in work when the rest of their family were working from home and yet we were coming to work every day. Uh, so there was, you know, we did do a lot there in terms of keeping people in touch with each other. When people came back to come from redeployment, we created supervision huddles so that we could get people to support each other with a very different way of working. I think. I think that was a particularly difficult time for staff because they've been whisked off into doing jobs that they've never done before and then when they came back to children’s speech and language therapy, it wasn't the children’s speech and language therapy that they'd left because it was a completely different, you know, scenario. So we put support in place for them.” (AHP Clinical Lead, A4_20)
- “And we lost. If you think of, but we lost 2 years’ worth of new workforce coming through because they couldn't continue with their training. And actually, what happened that some of the nurses, you know, and therapists that were within their training, left. They went back to their own country for a lot of them because they want it to be with their families and they've not come back.” (Commissioner, A3_07)

**Children and young people' health and well-being**

- “Biggest impact that we've seen with our children is, I've gone out on visits before when the equipment has become an essential need, but because they switched off physio services because there were redeployed. Some of our kids, if they had parent that was able and competent to do stretching and range of movement work great. But I also came across kids who hadn't had the physio, family wouldn't have done the physio and the child has got quite significant postural changes. So I'm thinking of your, CP children. That also results in a high demand on our service because those kids now need a different chair, a different wheelchair, they might need a sleep system on the bed.” (Equipment services, A4_14)
- “I think the biggest impact is on the spinal service. So with all the problems of accessing wheelchairs and stander, spines have got a lot worse and the waiting list now for spines is untenable. And so, [name], who’s our spinal surgeon. He’s got an urgent waiting list of 38 children, and there isn't space in PICU [pediatric intensive care unit]. And we've got children dying on the waitlist. And it’s awful. And, you know, I complain about my waiting list, but no one is actually dying.” (Paediatric Surgeon, A5_16)
- “Yeah, the knock on for our children with SEND, regardless of what their educational need, is reduce staff in school, reduce trusted adults in schools, the ones that they've got the relationship with either because they're poorly or because they're being pulled to cover a class, means that our kids are not accessing their learning. Even when they are in the classroom, they're more likely to dysregulate and as a consequence of that, we've got more exclusions. We got more part-time timetables, and we've got more going home educated.” (SEND Lead, A1_11)
- “'cause I think for some families it suited them very well having the lockdown. You know some of some of the autistic children quite liked being at home, even though it’s very challenging for their families. Then later on in the pandemic, I think for some of those autistic children with challenging behaviour, sleep difficulties, not being in their normal routine became a significant issue really and had an impact on their mental health.” (Paediatrician, A4_05)
- “So I think, short and long term I think there will be an increase in complexity and an increase in need. So particularly when we think around things like mental health issues, particularly anxiety, communication issues, child development, that kind of thing that’s, that will have been impacted.” (Commissioner, A3_01)

**Parent carer coping**

- “I think the whole experience for my families, has absolutely depleted their resilience and I think it has for some of my families really compounded that need of I cannot do this and I need my child to go residential. So throughout the pandemic I’ve had two children go residential but then, on top of that I’ve got a further three or four families, definitely three, four there’s one maybe teetering.” (Social worker, A5_11)
- “I think particularly all the preterm babies who, you know, already gone through quite a trauma and in hospital, those parents are then sort of doubly traumatised by being very isolated and not getting all the, not being able to show off their baby, not being able to see any other babies, discuss with other families and I think that’s been enormous for them and that’s ongoing, I think, still. And because their lungs will be vulnerable, they'll be worried that they'll pick up something. I think there are some good things that I think they've had to, they've just probably spent more time with their child and know they've had to deal with somethings. And then I think the biggest thing is probably isolation and lack of support being out there and available.” (Physiotherapist, A4_17)
- “So yeah, so we started off doing, Umm, sort of online groups. But actually we were finding that the take up was really low. So what we've done now is, it’s all online as webinars and parents are able to access them whenever they like… So I haven't seen the feedback, but it feels like it’s much better for parents because they're not having to take time off work to come at really specific times. And also it means that anyone who’s supporting the child can access them.” (Clinical psychologist, A3_03)

A few of the paediatricians and AHPs also suggested that the increased waitlists were partly a result of inappropriate referrals due to families not being seen by universal providers (eg, health visitors) and General Practitioners (GPs) during stages of the pandemic (box 2c). They received referrals that did not require specialist input and could have been supported by universal provision sooner if these services had been running as usual. Paediatricians and surgeons also commented that some children and young people who required specialist services presented much later. Medical professionals reported seeing delays in diagnosis or seeing differences in a child once they saw them in person compared with online. Where a diagnosis had been given just prior to or during the pandemic, there was a loss of follow-up during the pandemic. Referrals or signposting to support groups, workshops or other specialists could not happen; therefore, parents of children with a new diagnosis were left unsupported.

Transition care pathways were also said to be impacted by the reduction of in-person appointments. In healthcare, a couple of paediatricians explained that established transition clinics/processes continued, with some transition planning using virtual meetings. Whereas some of the education professionals described how children transitioning between schools could not attend the viewings of schools or meet new school staff, although some virtual tours were put in place. Social care transitions were also significantly affected (box 2d). Children’s social care professionals described the closure of adult social care and the lack of events for families to find out about post-18 years of age options.

Many of the professionals raised the challenges in managing safeguarding concerns during the pandemic (box 2e). Despite the sharing of data to identify vulnerable children and families, professionals across all sectors were concerned that some vulnerable children had slipped through the net with regards to safeguarding when they were not being seen in school or at in-person appointments. A couple of the social care professionals described the cumulative effects of families not receiving care and intervention. Where incremental deteriorations in children’s environments would have been picked up early and preventative measures could have been put in place before lockdown, these built up during the pandemic resulting in some children living in unacceptable environments, which became protection and safeguarding matters.

#### Workforce well-being

The adverse impacts of the pandemic, increased pressures and service disruption on staff well-being were reflected in the descriptions of burnout, feeling isolated and vulnerable while working remotely and lamenting the loss of camaraderie and support from their teams. All the team managers reported how they and their teams were working relentlessly, sometimes in times of fear and anxiety, trying to deliver the best care they could, but knowing there were limitations to what they could do. Team leads and managers described putting emotional and psychological support in place for their colleagues (box 2f). In all the sectors, at the time of the interview, many participants described how the burnout and changed job roles lowered job satisfaction and led to some teams losing the staff, meaning they now had gaps in the workforce in the recovery and resetting of services. Alongside this gap, a medical and an allied health professional was concerned that the changes to service delivery meant trainees had received less experience working directly with families and had missed training opportunities, leading to newly qualified staff feeling less confident in practice and needing more support in a period where the services were trying to recover (box 2g).

#### Children and young people’s health and well-being

Both medical and allied health professionals recognised that service changes had impacted children and young people’s physical health (box 2h,i). The negative impact of the loss of therapeutic services and physical checks was evident in children and young people’s deterioration in spasticity, dystonia, postural management and muscle contractures. For children with epilepsy, a couple of professionals reported a decrease in the occurrence of seizures potentially because children were not being overstimulated in school environments, whereas others suggested the reduction was due to families not reporting seizures out of fear their child would need to go into hospital. Both surgeons highlighted the devastating impact of delays in surgery on children and young people’s mobility, surgical outcomes, and how in some cases delays meant surgery was no longer possible.

All professionals observed behavioural changes, describing a decrease in dysregulated behaviour (eg, better sleeping patterns) for some children and young people who preferred being at home, while there was an increase in others because of being out of routine for significant periods of time (box 2j,k). A few of the education and social care professionals raised the difficulties some children were having with returning to school following so many periods of change. Children who had previously been ‘steady’ in school were returning with much greater needs, requiring more support and input. There were concerns about children and young people’s emotional and mental health, with many seeing higher levels of anxiety in children and young people related to the pandemic and increased referrals to Child and Adolescent Mental Health Services (CAMHS). Many thought the full repercussions of the pandemic on disabled children and young people’s physical and mental health were yet to be fully recognised and recorded (box 2l).

#### Parent carer coping

There was a wide recognition that the closure/restriction of services meant parent carers had taken on much, if not all, of the management of their child’s care and support during the pandemic. Commissioners and social care professionals discussed how the increased load on parent carers was evident by increased requests for short breaks provision and other support services (box 2m,n).

Professionals in each sector described seeing how the withdrawal of services and shift to increased or even 24/7 care for many parent carers had a devastating and deleterious impact on their well-being**,** exacerbating problems that existed prepandemic. Several medical and allied health professionals spoke about the well-being of the whole family, recognising the disabled child’s well-being could only be supported if the family unit was coping. Likewise, all the social care professionals reported seeing parent carers who were usually very stoic and resilient struggling with the added stressors of the pandemic.

A few professionals commented on how telephone calls with families became an opportunity for parent carers to talk to someone about how they were, or were not, coping. The professionals we interviewed recognised that parent carers could not access their usual peer support networks in place due to restrictions. A couple of the social care leads and a few of the AHP teams described trying to move parent carer groups online, but with mixed success due to parent carer engagement and parent carers being unable to prioritise their own needs to access support groups due to the pressures described above (box 2o).

### Learning for future service provision and emergencies

Participants reflected on what had been learnt from the pandemic in how services would recover and reset. As it was one of the biggest changes to service delivery, many professionals talked about the continued use of telehealth in each of their sectors (box 3a). Professionals across the sectors recognised that telehealth offered some flexibility in how families could choose to engage with and access services and had improved communication between teams around a child/family. Many medical and allied health professionals continued to offer a hybrid approach to appointments (offering option of the telephone, video or in-person appointments) and saw this continuing for the long term. The ongoing use of telehealth would allow for appointments to continue when a child or parent carer is too unwell to attend in person, when the child cannot leave home or when there is a concern that requires an immediate response. However, while there was a place for telehealth, it was highlighted by the majority that several activities need to be delivered in person to allow professionals to fully assess the children and deliver the necessary interventions. It was also recognised by all that telehealth does not work for all families or all services, with many children not able to engage in digital learning, for example. Telehealth was seen by many as an option to offer, but not a replacement for in-person activities. Participants stated that the continuation of effective telehealth would require investment in technology across services, as well as clear protocols and guidelines around the use of different platforms. Digital poverty and broadband access also needed to be addressed to allow equitable access to telehealth for all (box 3b).

Box 3 **Quotations for theme: learning for future service provision and emergencies**

- “So, I think it’s figuring out the balance[telehealth and in-person]and I do think that’s going to be different for every family. I think it’s about, what we need now is all those things that we've used over the pandemic. We want to keep them and make them as easy to use as possible and just have them as options. So, for some families you know, particularly if they're working or something like that, they don't want to be travelling to and from clinic spaces and stuff like that. So, it’s nice to have those options. So, I think it’s, it’s kind of embracing everything we did, just having it available and giving families those choices. And around what works for them.” (Occupational therapist, A3_05)
- “I suppose the other thing we haven't mentioned that is also a big thing that’s come out of the pandemic is understanding digital access and digital poverty. And for many families, being able to connect digitally is difficult because they don't have the data or they don't have, you know, they're just, it’s just not, it’s difficult and having that understanding about digital access is crucial and that we cannot assume that everybody is either able or willing to join meetings or do you know, to do clinical work with us, you know digitally and I think that’s obviously a really important thing that we have learned and you know really need to understand and that’s why choice is so important.” (Designated Medical Officer, A2_19)
- “We did a bit of service evaluation talking to adolescents around, how would you want to access services? And you know, it was around sensory needs, but it sort of became quite generic because we thought they'd all say I would just want a website or wanna do all online or whatever. And loads of the teenagers, so they just want to come and talk to someone face to face, actually. So that was like, that really influenced the way we set things up in the sensory service. And, you know, we didn't rush to just create online resources for teenagers. It was much, thinking much more about how can we give them what they're asking for, which was face to face stuff. And so that was really good.” (Therapies Lead, A3_02)
- “So what happened was, in the early stages of that first lockdown, I was contacted by one of the Commissioners for Children to say, how are you meeting the needs of children with special educational needs? And from that, it developed into a monthly meeting that we have for children’s integrative therapies, physio, OT and speech and language therapy with strategic leads from [organisation], the local authority and the CCG, and that’s been fantastic, because now we've created all sorts of new kind of links and that’s really helped in terms of the messages, because if we had something that we wanted shared with all education settings, we could send it to them and we knew that it had gone to everybody and vice versa. So we're now regularly attending SENDCo forums and. So it’s created a much more collaborative working than that we had in the past.” (Therapies lead, A4_20)
- “Half the time, I think we're at the minimal amount already. I wouldn't want it to drop. I'd have twice as many doctors and I'd have quite a few specialist nurses, thank you very much. And a few clinical psychologists and we’d be just laughing… But I wouldn't want to do less than we did. Yeah. Don't think it would be safe somehow.” (Designated Medical Officer, A1_03)

Some medical and allied health professionals reported that the pandemic had made them reflect on child/family-centred approaches, for example, using hybrid approaches to appointments if they work well for the family and child (box 3c). It was recognised that travelling for in-person appointments could be challenging for some families due to, for example, journey time, the child’s needs or other commitments (eg, work, school and other children). Experiences during COVID-19 have indicated the importance of offering families more choices in how they engaged with services and vice versa. Others described the pandemic highlighting the importance of taking a needs-led rather than diagnosis-led approach to ensure intervention was focused on the areas the child or young person needed support in. Some spoke about how changes during COVID-19 have allowed them to refocus on what the family and child want from service input and what is important to them.

The pandemic has led to networks working collaboratively, as described by the RAG rating above. Many wanted this collaborative working to continue and identified the need for data sharing (such as that allowed when the COPI notice was in place). Commissioners thought there should be a reflection on the experience and learning during the pandemic to recognise the efficiencies and benefits of data sharing and the way this could centre support on the individual child (box 3d). Commissioners and service leads also recognised the importance of meaningfully involving parent carers and disabled children and young people in the design of services going forward.

All interviews ended with a question about minimally acceptable service provision during a future emergency. All participants struggled to answer this question, with some commenting that they felt services were already at a minimum before the pandemic, and that if families have a need, it should be addressed adequately (box 3e). Table 2 indicates the thoughts of participants in the different groups on minimal service provision. Overall, children and families need to be kept safe, in relation to health and safeguarding, and care should start from the assumption that in-person support is needed until they are confident children and young people are safe and can manage with remote care.

**Table 2:** Views of different professional groups about minimally acceptable level of service provision

Medical clinicians /professionals reported	Allied health clinician/professionals reported
Maintain some in-person care where medically necessary Safeguarding in acute paediatrics needs to continue Keep specialist professionals in their job roles (not redeployed) Use telehealth to maintain continuity of care School closures as a last resort Proactive approach for services checking on families	Need to maintain in-person care where necessary for health outcomes, for example, under 5s, children with equipment and handling needs, Special provision schools should be kept open Short breaks services should remain open Data sharing across services to have a holistic profile of the child and their needs Enough intervention to allow families to cope in a crisis Enough staff capacity to continue to fulfil EHCPs

Education and social care reported	Commissioners reported
Whatever keeps the child and family safe Need effective online education offer for disabled children Social care—start from an assumption that in-person contact is needed until they are confident that children are safe and can manage with more remote care. Short breaks provision should continue in some capacity	Must be someone to contact in all services; there must be someone to talk to High level leadership across health and other agencies that was driving at a national and strategic level Duty of care to maintain safeguarding quality of services Schools remain open Digital offer for families

EHCPEducation, Health and Care plan

## Discussion

Rapidly implemented regulations to contain COVID-19 in England and guidance on their implementation led to many services to disabled children being deprioritised as they were seen as ‘non urgent’ or ‘non-essential’. The accounts of the professionals interviewed indicated the considerable pressure that health, education and social care services and staff were under to reconfigure the delivery of care in a rapidly changing context. However, this study has found that within the guidance, decisions were made at the local level and within teams on which needs were prioritised, how care was delivered and how the workforce was reorganised to achieve this. Participants described guidance being interpreted differently across the sectors, and an integrated approach to care for disabled children during the emergency was perceived to be lacking at a national level. However, the change in legislation under the COPI notice did allow health, education, and social care teams to share information on individual children and families, which was described as enabling holistic, individualised care within the confines of a reduced service and health protection measures, such as social distancing and PPE.

Care providers reported concerted efforts to communicate with families during the pandemic, in the knowledge that they were under significant strain as sole care providers with extremely limited formal or informal support. The rapid implementation of telehealth was perceived to have worked well for the medical management of known conditions but described as less successful for the assessments and interventions that require physical involvement and engagement of the child, including many AHP interventions. All participants recognised that the changes in service provision had not worked for families and there had been a lack of preparedness for such an emergency that had resulted in significant negative impacts on children, young people and their families. While best efforts had been made to reach families and support them within the guidance provided, there was recognition that some families’ and young people’s needs were missed or not met. Interviews with parent carers and young people as part of this research programme indicated that communication about service change and access to needed support was not sufficient during the COVID-19 pandemic leading to long-lasting impacts on both parent carer and young people’s health and well-being.^15^

The perspectives reported are those of health, education and social care professionals working with disabled children. Interviews with commissioners, managers and frontline workers provide an overview of how decisions were made, how they were implemented and how well they worked during the stages of the pandemic. The timings of the interviews (November 2021 to September 2022) allowed for reflection on what had been learnt from earlier stages of the pandemic and the continued challenges faced by services as national restrictions were gradually removed. Although, this also means that interviews were collecting post hoc accounts of decisions and experiences, which may be recalled and reflected on differently at this time point compared with the actual time of events. Limitations of this study include potential response biases. While efforts were made to recruit a wide range of health, education and social care professionals in each area, there was variation in the types of professions interviewed in each area. In terms of potential response bias, our interviewees may be a more engaged subgroup of professionals who were motivated to participate than their colleagues and may have had a particular personal perspective. There may also be professionals who will have opted not to participate in this research due to the potentially triggering nature of discussing their experiences while working during the COVID-19 pandemic.

This paper reports the overall experience of professionals across five diverse areas of England. We did not seek to make comparisons of experiences between the different areas with their different characteristics (eg, rural vs urban, having a specific children’s NHS trust). This would require sufficient contextual and granular data which could further elaborate on learning for future emergencies and recovery, for instance how health and social inequalities in the UK were exacerbated by the COVID-19 pandemic is increasingly acknowledged. Further to this, the experiences of parent carers and young people are presented in a separate paper in order to be able to present both perspectives in the necessary detail needed, given the comprehensive data we were able to collect from both groups.

The challenges in providing services in the rapidly changing landscape of the COVID-19 pandemic, accessing appropriate PPE, getting clear guidance and the lack of preparedness described by the participants reflect the findings from other work describing the experience of health, education and social care professionals in England.^21 22^ The study findings mirror those of other European nations, North America and Australia, where social distancing measures led to reductions in access to services and the implementation of telehealth which, on the whole, worked better for medically managed conditions than those led by AHPs.^23^,^28^ Many professionals across each sector saw a place for telehealth going forward, but better access, protocols, tools and training for delivering telehealth continue to be needed to embed this as an effective tool for service delivery. However, digital healthcare is not accessible to all families and may exacerbate existing health inequalities,^29^,^31^ further highlighting the need for an individualised approach to disabled children’s care.

Existing prepandemic waitlists and reduced staffing and restricted service delivery during COVID-19 lockdowns and restrictions resulted in extensive wait times leaving parents to manage complex physical and behavioural needs. This situation was reflected across the NHS and its links with social care whereby prepandemic weakness exposed vulnerable populations to further inequalities and avoidable harms.^32^ Continuity of universal services in emergencies and consistent systems for prioritisation of high-risk children are necessary to ensure accurate triage of children and their needs and a means of providing intervention to prevent long-term detrimental impact on young people and families.

There was a pressing need to support the health needs of parent carers of disabled children before the pandemic.^33^ The profound and prolonged additional pressure from service disruption compounded the problems. Addressing the health of carers is already a priority for the NHS;^34^ the health needs of parent carers must not be neglected in the recovery from the COVID-19 pandemic or forgotten in any future emergencies. Upscaling specific parent carer-focused health promotion programmes such as Healthy Parent Carers offer considerable potential for addressing the recognised risks of physical and mental health problems.^35^ The impact on parent carers and young people is described in more detail in a parallel paper from this programme of research.^15^ Findings of both studies and our previous scoping review mapping the international literature^5^ informed the development of a set of recommendations on service commissioning and provision in future emergencies through a national consensus survey as the final part of this commissioned research.

The cumulative effects of cuts to services prepandemic,^36^ long-standing failures to effectively integrate care,^37^ the impact of the pandemic on staff well-being and reductions in workforce,^38 39^ have made the recovery and resetting of services challenging. Concerns prevail about the long-term effects on the mental health of health professionals from their experiences on the frontline during the pandemic.^40 41^ The pandemic highlighted and, in some cases, amplified the existing challenges and inequalities within the system.^42 43^ Many professionals described the pressure to ‘return to normal’ while working in a system that has failed to embrace the flexibility and innovation that was necessary and permitted during the pandemic. Analysis of what worked during the pandemic should be informing services and enabling them to provide more diverse and innovative means of service access.^44^

### Conclusion

Local teams acted innovatively to interact with and continue to support and maintain health, education and social care provision to disabled children and their families during the COVID-19 pandemic. Telehealth enabled some continuity of care but not all assessments and interventions were possible, leading to the exacerbation of health needs, safeguarding concerns, but also of existing inequalities for families of disabled children. Service reduction increased the already long waitlists of new children and known children with new needs. This and the longevity of the pandemic have had negative consequences for the health and psychosocial outcomes of children, young people, parent carers and their families and professional’s health and well-being.

The redeployment of staff from children’s services, the closure of schools and the lack of clear guidance for working with disabled children and their families indicated how this group was not prioritised during the COVID-19 pandemic. The needs of disabled children, parent carers and their families must be prioritised in planning for future emergencies and the recovery of services. Key learning from this study is the need to quickly identify disabled children and their level of need and risk, assess the impact of any reduction or loss of services, clear and consistent guidance across sectors and services taking into account the complex needs of many disabled children and to work collaboratively with families to develop child-centred care to provide more resilience during service disruption.

## supplementary material

10.1136/bmjopen-2024-085143 online supplemental file 1

10.1136/bmjopen-2024-085143 online supplemental file 2

## Data Availability

Data are available upon reasonable request.
