# Supplementary material for: Professional perspectives on facilitators and barriers for high quality provision of health, education and social care services to disabled children in England during the COVID-19 pandemic: a qualitative study
Source: BMJ Open. 2024 Aug 24;14(8):e085143. doi: 10.1136/bmjopen-2024-085143 (PMC11733913; doi:10.1136/bmjopen-2024-085143)
Supplement: online supplemental file 1 [file bmjopen-14-8-s001.pdf]

Table 1: Patient and Public Involvement and Engagement Activity

**Section and topic**	**Item**
1: Aim	The aim of PPIE engagement was to ensure relevance and reliability of this research study. Children and young people with neurodisability and their parent carers provided a unique perspective on the changes to their services and lived experience of the impact of these changes.
2: Methods	Three groups were involved:<br>1. Peninsula Childhood Disability Research Unit Family Faculty parent carer group.<br>2. A project specific parent carer advisory groups were recruited through parent advocacy organisations.<br>3. A young person advisory group were recruited through specialist schools local to the to the researchers University.<br><br>Each group met regularly at each stage of the research to review methodologies, recruitment processes and to review findings and our interpretation. Parent carer advisory groups met four times, and young people's groups met four times. The advisory groups were initially asked to provide feedback on the interview tools. Later, groups reviewed the gaps identified by the Mapping Review. Meeting materials were tailored to enable access to all group members including those with communication support needs. In the final meetings in each group, the interview findings were shared and discussed.
3: Study results	The advisory group helped with updating study information sheets and in particular advised on recruitment strategies for the young people and parent carers. The advisory groups provided validation and additional context to the interpretation of the interview findings.
4: Discussion and conclusions	PPI engagement confirmed the impact of service changes on families. This contribution directly informed the study and recommendations developed in the next stage of the research.
5: Reflections/critical perspective	Young people contributing to the PPI suggested further opportunities to engage with the process this included providing an accessible overview of the study structure and timeline with clear visual identification and mapping of the PPIE process. Young people's PPI engagement gave insight into some of the challenges young people may face in involving in interviews, something which could be factored in earlier in project design.
