# Supplementary material for: Professional perspectives on facilitators and barriers for high quality provision of health, education and social care services to disabled children in England during the COVID-19 pandemic: a qualitative study
Source: BMJ Open. 2024 Aug 24;14(8):e085143. doi: 10.1136/bmjopen-2024-085143 (PMC11733913; doi:10.1136/bmjopen-2024-085143)
Supplement: online supplemental file 2 [file bmjopen-14-8-s002.pdf]

Resetting Services to Disabled Children

Interview Topic Guide: Professionals

Introduction

Aim of the project is learn from the changes made in response to COVID-19 and find out how services for disabled children could be remodelled to be effective and acceptable. We want to hear from providers about the changes made to service, what worked and what didn't. There aren't any right or wrong answers. Anything you tell us will be confidential. If we use quotes from interviews in reports, they won't be attributable to an individual, an organisation or an area.

Consent to record the interview

You've previously been sent information about the project and filled in the consent form; thank you for doing that. Are you still happy for us to record the interview?

[Group interviews] We really want to hear from everyone. Sometimes it's difficult to see if someone is waiting to speak on group calls, so do raise your hand to let us know you want to say something. We'll all try not to speak if anyone else is talking so we can hear everyone on the recording. While we will keep your participation confidential and will not attribute anything said in this interview to any participant, we cannot prevent participants from discussing this with others. Please be respectful of the privacy of fellow participants and refrain from divulging anything about other participants or things they have disclosed to people who are not present here.

Interview questions

Briefly and in turn, could you introduce yourself and say your job role?

[remind participants keep this short]

Thinking back to March 2020, when the first lockdown started, what changed about the service?

Probe:

1. Where: setting of care (e.g. outreach, inpatient to outpatient), remote working, how standard in-person care was managed; telemedicine
2. Coordination of care and management of care process: integration, shared care processes, transition of care,
3. How and when care was delivered: queuing, triage, coordination across providers of care,
4. Who delivered care and how that was managed: role change/expansion, patient self-management, frequency of consultations.

medium of provision, personnel delivering care, timescale of changes, role changes, remote working,

Immediate changes made vs developments over the year as we moved in and out of lockdowns.

What influenced the decisions about what and how to change?

Probe: clinical need (urgency), processes (policy/protocol/safeguarding/continuity of care), resources – staff, digital, equipment etc, were they classified as an essential service?

What were the barriers or facilitators to implementing change?

For staff, families. Why? Costs, facilities, staff training

What worked well? Why?

Probe: for you, families, organisations providing services e.g. parental involvement in therapy programmes
Impact on changes in health and wellbeing of children and family

Who did the changes not work well for? Why is this?

Probe: child, family, environment factors

Impact of changes on health and wellbeing of children, family

Any groups left behind? Why?

What worked well for these groups before the pandemic?

Are any activities difficult to complete in the changed model of delivery

Assessment - of what?

Intervention / management - of what?

Safeguarding

What impact have the changes had on multidisciplinary working? (Inc. cross-sector teams)

Impact of changes on professionals and organisations (Probe: short and longer term)

Going forward, what should we

Continue?

Stop?

Start?

Close

Thank participants

Next steps: interviewing other families and providers of services, bring information together what has and hasn't worked for which groups and why. Future survey to agree recommendations. Will contact family about that using details they previously supplied. Family can get in touch any time about the study. Updates will be on website.
