# Supplementary material for: Professional perspectives on facilitators and barriers for high quality provision of health, education and social care services to disabled children in England during the COVID-19 pandemic: a qualitative study
Source: BMJ Open. 2024 Aug 24;14(8):e085143. doi: 10.1136/bmjopen-2024-085143 (PMC11733913; doi:10.1136/bmjopen-2024-085143)
Supplement: Supplementary file 3 [file bmjopen-2024-085143.reviewer_comments.pdf]

PEER REVIEW HISTORY

BMJ Open publishes all reviews undertaken for accepted manuscripts. Reviewers are asked to complete a checklist review form (**http://bmjopen.bmj.com/site/about/resources/checklist.pdf**) and are provided with free text boxes to elaborate on their assessment. These free text comments are reproduced below.

ARTICLE DETAILS

**TITLE (PROVISIONAL)**	Professional perspectives on facilitators and barriers for high quality provision of health, education and social care services to disabled children in England during the COVID-19 pandemic: a qualitative study.
**AUTHORS**	Merrick, Hannah; Driver, Helen; Main, Chloe; Potts, Lily; Russell, Sian; Exley, Catherine; Allard, Amanda; Morris, Christopher; Parr, Jeremy; Pennington, Lindsay; Resetting Services Group, .

VERSION 1 – REVIEW

**REVIEWER**	Archard, Philip John<br>Tavistock and Portman NHS Foundation Trust, Education and Training
**REVIEW RETURNED**	23-Apr-2024

**GENERAL COMMENTS**	I thought this was a valuable paper that will be of interest to health and social care researchers and professionals. It reports of an ambitious study, which provides something of a wide scope analysis. Overall, it is well written and appropriately presented.<br><br>I had some thoughts about how it may be further developed, but I appreciate the authors are working with specific limitations in attempting to report the work in a succinct way.<br><br>I wondered if more could be said about ethical considerations relating to the research and participation of professionals (who were potentially adversely affected during the pandemic and asked to speak about their experiences). I also thought the analysis could have been more fine grained at times, i.e., there are no 'counts' of the number of participants who made reference to a specific issue, which makes it harder for the reader to discern how much a claim made is representative of all, some or a few of accounts of represented in the analysis. A good command of the data set would suggest that some references of this kind can be included in the analysis.<br><br>Regarding the method/methodology, I wondered if there was something of a drift towards language associated with quantitative research, which may not apply (in the same way) to this study as qualitative research, i.e. reference to objectivity (is it not assumed this is a situated understanding and there is no 'view from nowhere'?) and results (would 'findings' be preferable?). More could also be said about the rationale for the specific number of participants recruited and, though it does make sense, why this analysis of professional accounts was reported separately from the broader research project (or interviews with parents/carers).

	Discussion: I'm unsure the 'main finding' expressed at the beginning of the discussion is the 'main finding' of the study so much as something that was evidenced in the accounts of the participating professionals; it is more that discretion was exercised about what to prioritize at a local level amidst considerable pressures on services and staff. I'd be more cautious in the way the interpretations of what the participants said is reported. At present, it reads as though what they said was factually accurate. I'm not saying they don't represent the reality of the work at this time, but they, are all the same, their perspectives which may – or may or not – concur with the accounts of professionals in other services (and observations of actual working practices during this period).<br><br>I think there's perhaps more to be said about the implications of the pandemic experience for service planning and policy more generally. What is touched on here, though not elucidated more fully, is the ways in which, though the pandemic had a significant impact on service delivery that was detrimental, there was also innovation in specific contexts, and the experience at this time seemed to orientate many professionals and public healthcare systems to a more compassionate attitude or 'intelligent kindness' in services (see Damsgaard, J. B., & Phoenix, A. (2022). World of Change: Reflections within an educational and health care perspective in a time with COVID-19. International Journal of Social Psychiatry, 68(1), 177-182). The issue of staff wellbeing and links to moral injury is also perhaps something that can be acknowledged in the discussion (see, e.g., Lesley, M. (2021). Psychoanalytic perspectives on moral injury in nurses on the frontlines of the COVID-19 pandemic. Journal of the American Psychiatric Nurses Association, 27(1), 72-76).<br><br>I wonder if more could be said in the discussion section by, briefly, comparing the findings of this study other studies that have examined the experiences of UK health and social care professionals during the pandemic, and their experience of serving (disabled) children and their families amidst shifting expectations of the 'help' that can be provided.<br><br>More minor issues:<br>I'd say the whole paper needs a thorough proofread and copyedit. It is well written and comprehensible but there are inconsistencies, i.e. in COVID, Covid, covid. The quoted transcripts also don't seem to be entirely consistent in terms of the way numbers are expressed, terminology etc.<br>Language: disabled children or children with disabilities?<br>p. 3. 'important part' or 'important role'<br>p.9 'and in some areas, participants described teams being reduced from ten to two members of staff because of redeployment which decimated the capacity of that service.': some different area teams experienced the same reduction in staffing numbers (which is what this statement suggests)?

**REVIEWER**	Shakespeare, Tom<br>London School of Hygiene and Tropical Medicine Faculty of Infectious and Tropical Diseases
**REVIEW RETURNED**	23-Apr-2024

**GENERAL COMMENTS**	This is a good clear, welll researched paper that could I think be published "as is".

	From the title, I was expecting to hear about professionals' experiences: instead, it is about service changes as a result of Covid-19. So perhaps title could be made more accurate to paper.<br><br>What did professionals get wrong? To read this, not much. But clearly, not all went well. Mayube refer more to liteature about impact of pandemic on disabled adults and children. Our own data found: a lack of contact from social care services; disabled children's access to education being an afterthouight (after nondisabled children); lack of rehabiitation contact (e.g. community physio talking about how she could only stand at garden gate and shout instructions on exercises etc for child with cerebral palsy). Perhaps these type of limitations and failures could have more space?<br><br>But maybe these are about emphasis. Paper otherwise good.

VERSION 1 – AUTHOR RESPONSE

Reviewer: 1
Dr. Philip John Archard, Tavistock and Portman NHS Foundation Trust
Comments to the Author:
I thought this was a valuable paper that will be of interest to health and social care researchers and professionals. It reports of an ambitious study, which provides something of a wide scope analysis. Overall, it is well written and appropriately presented.
Thank you.
I had some thoughts about how it may be further developed, but I appreciate the authors are working with specific limitations in attempting to report the work in a succinct way. I wondered if more could be said about ethical considerations relating to the research and participation of professionals (who were potentially adversely affected during the pandemic and asked to speak about their experiences).

Thank you. We have added some additional information on the ethical considerations relating to the research. On page 4 we have added: “Given the sensitive nature of the interview and topics being discussed, it was explained to all participants that they could stop the interview at any point they needed to and did not need to answer a question if they did not wish to.”
On page 20 we have added: “There will be professionals who will have opted not to participate in this research due to the potentially triggering nature of discussing their experiences while working during the COVID-19 pandemic.”

I also thought the analysis could have been more fine grained at times, i.e., there are no ‘counts’ of the number of participants who made reference to a specific issue, which makes it harder for the reader to discern how much a claim made is representative of all, some or a few of accounts of represented in the analysis. A good command of the data set would suggest that some references of this kind can be included in the analysis.

Thank you. We have gone through the results section to provide more detail on the representative of claims. Counting in qualitative research is controversial due to sampling. Therefore, we have not added counts as such, however we have indicated where issues were raised by a more, or the majority, all, or a single member of specific groups of the participants interviewed.

Regarding the method/methodology, I wondered if there was something of a drift towards language associated with quantitative research, which may not apply (in the same way) to this study as

qualitative research, i.e. reference to objectivity (is it not assumed this is a situated understanding and there is no ‘view from nowhere’?) and results (would ‘findings’ be preferable?).

Results is the preferred heading of the journal for the paper section, however elsewhere in the manuscript we have referred to ‘findings’. We appreciate the reviewer’s point however and have edited the description of our reflexivity section to be more in-line with qualitative methodology – page 4 “Researchers were conscientious in reporting the narrative of participants, engaging in reflection and discussion to maintain objectivity ensure accurate interpretation of the interview findings.”

More could also be said about the rationale for the specific number of participants recruited and, though it does make sense, why this analysis of professional accounts was reported separately from the broader research project (or interviews with parents/carers).

Thank you. We have added a sentence to page 4 to describe the rationale for the sample size.
“We aimed to purposively sample approximately 15 multidisciplinary professionals per area to allow us to capture a range of different views.”
We elected to publish this work as a pair of papers so that the appropriate space could be given to each perspective to reflect the rich and comprehensive data that was collected from both professionals and families. The papers have been submitted to the same journal as sister papers and we hope will be published alongside each other. We have added detail of this to the discussion on page 20.
“Further to this, the experiences of parent carers and young people are presented in a separate paper in order to be able to present both perspectives in the necessary detail needed, given the comprehensive data we were able to collect from both groups.”

Discussion: I’m unsure the ‘main finding’ expressed at the beginning of the discussion is the ‘main finding’ of the study so much as something that was evidenced in the accounts of the participating professionals; it is more that discretion was exercised about what to prioritize at a local level amidst considerable pressures on services and staff. I’d be more cautious in the way the interpretations of what the participants said is reported. At present, it reads as though what they said was factually accurate. I’m not saying they don’t represent the reality of the work at this time, but they, are all the same, their perspectives which may – or may or not – concur with the accounts of professionals in other services (and observations of actual working practices during this period).

Thank you. The first paragraph of the discussion has been edited to better reflect the main findings of this study and to ensure it reflects what was reported by our participants and not as factual.

“Rapidly implemented regulations to contain COVID-19 in England and guidance on their implementation led to many services to disabled children being deprioritised as they were seen as ‘non urgent’ or ‘non-essential’. The accounts of the professionals interviewed indicated the considerable pressure that health, education and social care services and staff were under to reconfigure delivery of care in a rapidly changing context. However, this study has found that within the guidance, decisions were made at the local level and within teams on which needs were prioritised, how care was delivered and how the workforce was reorganised to achieve this. Participants described guidance being interpreted differently across sectors and an integrated approach to care for disabled children during the emergency was perceived to be lacking at a national level. However, the change in legislation under the COPI notice did allow health, education, and social care teams to share information on individual children and families, which was described as enabling holistic, individualised care within the confines of a reduced service and health protection measures, such as social distancing and PPE. Care providers reported concerted efforts to communicate with families during the pandemic, in the knowledge that they were under significant strain as sole care providers with extremely limited formal or informal support. The rapid

implementation of telehealth was perceived to have worked well for medical management of known conditions but described as less successful for assessment and interventions that require physical involvement and engagement of the child, including many AHP interventions."

I think there's perhaps more to be said about the implications of the pandemic experience for service planning and policy more generally. What is touched on here, though not elucidated more fully, is the ways in which, though the pandemic had a significant impact on service delivery that was detrimental, there was also innovation in specific contexts, and the experience at this time seemed to orientate many professionals and public healthcare systems to a more compassionate attitude or 'intelligent kindness' in services (see Damsgaard, J. B., & Phoenix, A. (2022). World of Change: Reflections within an educational and health care perspective in a time with COVID-19. International Journal of Social Psychiatry, 68(1), 177-182).

Thank you. More detail has been added to the 'service delivery arrangements' section on page 9 to describe some of the innovative practices that teams described. A quotation has also been added to Box 1 to support this.
We have also included the suggested reference to page 21 in the final paragraph before the conclusions. "Analysis of what worked during the pandemic should be informing services and enabling them to provide more diverse and innovative means of service access {Damsgaard, 2022 #42}."

The issue of staff wellbeing and links to moral injury is also perhaps something that can be acknowledged in the discussion (see, e.g., Lesley, M. (2021). Psychoanalytic perspectives on moral injury in nurses on the frontlines of the COVID-19 pandemic. Journal of the American Psychiatric Nurses Association, 27(1), 72-76).

Thank you. On page 22 we have added: "Concerns prevail about the long term effects on the mental health of health professionals from their experiences on the frontline during the pandemic {Lesley, 2021 #41}.

I wonder if more could be said in the discussion section by, briefly, comparing the findings of this study other studies that have examined the experiences of UK health and social care professionals during the pandemic, and their experience of serving (disabled) children and their families amidst shifting expectations of the 'help' that can be provided.

Thank you. We have added the following to the discussion with some additional references to compare our findings to others in the UK.
"The challenges in providing services in the rapidly changing landscape of the COVID-19 pandemic, accessing appropriate PPE, getting clear guidance, the lack of preparedness described by the participants reflects the findings from other work describing the experience of health, education and social care professionals in England (21, 22)"

More minor issues:
I'd say the whole paper needs a thorough proofread and copyedit. It is well written and comprehensible but there are inconsistencies, i.e. in COVID, Covid, covid. The quoted transcripts also don't seem to be entirely consistent in terms of the way numbers are expressed, terminology etc.

Thank you. We have gone through the manuscript and ensured COVID is consistently presented and we have reviewed the quotations for consistency.

Language: disabled children or children with disabilities?
We appreciate conflicting view on this terminology, we prefer to use 'disabled children' to be

consistent with the social model of disability, that the person is disabled by their environment. Nevertheless, we appreciate alternate views prevail on 'person first' terminology.

p. 3. 'important part' or 'important role'

Thank you we have edited accordingly.
p.9 'and in some areas, participants described teams being reduced from ten to two members of staff because of redeployment which decimated the capacity of that service.': some different area teams experienced the same reduction in staffing numbers (which is what this statement suggests)?

Thank you, this sentence has been edited to ensure there is clear meaning on page 8.
"In one area plans for redeployment were not implemented, in another area redeployment was voluntary, and in remaining areas, AHP participants described their teams being reduced significantly (for example one physiotherapy team was described as going from ten to two members of staff) because of redeployment which decimated the capacity of that service."

Reviewer: 2
Prof. Tom Shakespeare, London School of Hygiene and Tropical Medicine Faculty of Infectious and Tropical Diseases
Comments to the Author:
This is a good clear, well researched paper that could I think be published "as is".
Thank you.

From the title, I was expecting to hear about professionals' experiences: instead, it is about service changes as a result of Covid-19. So perhaps title could be made more accurate to paper.

Thank you for highlighting this with regards to our title. This has now been changed to the following: A qualitative study to identify facilitators and barriers for high quality provision of health, education and social care services to disabled children in England during the COVID-19 pandemic

What did professionals get wrong? To read this, not much. But clearly, not all went well. Maybe refer more to literature about impact of pandemic on disabled adults and children. Our own data found: a lack of contact from social care services; disabled children's access to education being an after thought (after nondisabled children); lack of rehabilitation contact (e.g. community physio talking about how she could only stand at garden gate and shout instructions on exercises etc for child with cerebral palsy). Perhaps these type of limitations and failures could have more space? But maybe these are about emphasis. Paper otherwise good.

The interviews with professionals were focussed on how decisions were made and how services changed. We asked professionals to reflect on what can be learned for the future but did not focus on what they got wrong as such. We have added some more detail to the discussion to emphasise that professionals recognised that the changes did not work well for families (page 19), as is also reflected in the findings on the impacts of the service change.
"All participants recognised that the changes in service provision had not worked for families and there had been a lack of preparedness for such an emergency, that had resulted in significant negative impacts on children, young people, and their families. While best efforts had been made to reach families and support them within the guidance provided, there was recognition that some families' and young people's needs were missed or not met. Interviews with parent carers and young people as part of this research programme, indicated that communication about service change and access to needed support was not sufficient during the COVID-19 pandemic leading to long-lasting impacts on both parent carer and young people's health and wellbeing {Merrick, submitted #2}."

Our other paper (referenced above), describing the parent carers and young people's experience of service change during the pandemic reflects more what services got wrong and what did not work for families.

VERSION 2 – REVIEW

**REVIEWER**	Archard, Philip John<br>Tavistock and Portman NHS Foundation Trust, Education and Training
**REVIEW RETURNED**	13-Jun-2024

**GENERAL COMMENTS**	I appreciate the thoroughgoing and thoughtful response the authors have provided to the comments made during the initial round of peer review. The paper was, I think, strong at first submission and these revisions have helped refine it. I think it is now publishable in its present form (or at least there is nothing more I would want to add as reviewer at this stage).<br><br>For what its worth, and issues that may be picked up and addressed at the copyediting stage, I noticed.<br>The second and fourth points after the abstract are unclear and could be revised.<br>I'm unsure about the language of 'triggering' on p.20. I wonder if describing this in terms of how people were affected would be preferable and give more of an impression of a range of painful experiences (rather than assumed traumatisation).<br>There are a few minor issues that should be picked up re precise wording and grammar, e.g, reference to 'school nursers' and a non-sentence beginning 'For example,..' on p.9.<br>I hope these comments are helpful to the authors.

VERSION 2 – AUTHOR RESPONSE

Reviewer: 1
Dr. Philip John Archard, Tavistock and Portman NHS Foundation Trust
Comments to the Author:
I appreciate the thoroughgoing and thoughtful response the authors have provided to the comments made during the initial round of peer review. The paper was, I think, strong at first submission and these revisions have helped refine it. I think it is now publishable in its present form (or at least there is nothing more I would want to add as reviewer at this stage).
For what its worth, and issues that may be picked up and addressed at the copyediting stage, I noticed.
The second and fourth points after the abstract are unclear and could be revised.
We have reworded points 2 and 4 below the abstract to ensure they are now clear. Thes now read as:
• Qualitative methodology enabled in-depth data collection about reflections on service changes and decision making.
• Framework analysis enabled inductive thematic analysis and data exploration whilst simultaneously maintaining a transparent audit trail.

I'm unsure about the language of 'triggering' on p.20. I wonder if describing this in terms of how people were affected would be preferable and give more of an impression of a range of painful experiences (rather than assumed traumatisation).
We have altered the wording of 'triggering' in the discussion on page 19 (previously page 20). This

has been altered to: “There may also be professionals who will have opted not to participate in this research due to how they were affected by the pandemic (both in their professional and personal experience) and not wishing to discuss and reflect on their experiences while working during the COVID-19 pandemic for the study.”

There are a few minor issues that should be picked up re precise wording and grammar, e.g, reference to 'school nursers' and a non-sentence beginning 'For example,..' on p.9.

We have gone through the manuscript to revise any formatting issues. Including correcting ‘school nursers’ to ‘school nurses’.

Thank you for the opportunity to make these revisions.

Best wishes,
Dr Hannah Merrick
on behalf of the Resetting Services Team.
